# Pharmacogenetic analysis of structural variation in the 1000 genomes project using whole genome sequences

**DOI:** 10.1038/s41598-024-73748-3

**Published:** 2024-10-01

**Authors:** Carissa A. Sherman, Katrina G. Claw, Seung-been Lee

**Affiliations:** 1 Department of Biomedical Informatics, Colorado Center for Personalized Medicine, University of Colorado Anschutz Medical Campus, Aurora, CO USA; 2 Macrogen Inc, Seoul, Republic of Korea

**Keywords:** Pharmacogenetics, Population genetics, Structural variation, Star alleles, Next-generation sequencing, Haplotypes, Population genetics, Sequencing

## Abstract

While significant strides have been made in understanding pharmacogenetics (PGx) and gene-drug interactions, there remains limited characterization of population-level PGx variation. This study aims to comprehensively profile global star alleles (haplotype patterns) and phenotype frequencies in 58 pharmacogenes associated with drug absorption, distribution, metabolism, and excretion. PyPGx, a star-allele calling tool, was employed to identify star alleles within high-coverage whole genome sequencing (WGS) data from the 1000 Genomes Project (N = 2504; 26 global populations). This process involved detecting structural variants (SVs), such as gene deletions, duplications, hybrids, as well as single nucleotide variants and insertion-deletion variants. The majority of our PyPGx calls for star alleles and phenotype frequencies aligned with the Pharmacogenomics Knowledge Base, although notable population-specific frequencies differed at least twofold. Validation efforts confirmed known SVs while uncovering several novel SVs currently undefined as star alleles. Additionally, we identified 210 small nucleotide variants associated with severe functional consequences that are not defined as star alleles. The study serves as a valuable resource, providing updated population-level star allele and phenotype frequencies while incorporating SVs. It also highlights the burgeoning potential of cost-effective WGS for PGx genotyping, offering invaluable insights to improve tailored drug therapies across diverse populations.

## Introduction

Pharmacogenetics (PGx) explores links between genetic variations and drug responses, offering potential optimization of medication selection, dosage, and efficacy^1^. Its capacity to mitigate adverse drug events, encompassing potentially harmful reactions or side effects, stands as a primary concern for global health systems^2^. There are growing efforts to characterize global PGx variation to address the current lack of reliable haplotype frequencies across diverse populations.

The “star allele” nomenclature system is used in PGx to standardize genotypes (e.g., CYP2D6*4) with predicted clinical phenotypes (e.g., poor metabolizer)^3^. There are exceptions to this nomenclature; for example, the *G6PD* gene uses its own system^4^. The principle remains to match genetic data (diplotype calls such as CYP2D6*2/*4) with predicted phenotypes based on translation tables stored in PGx databases. Despite its utility, accurately measuring star alleles faces challenges in detecting rare variants^5^ and structural variants (SVs) like gene deletions, duplications, and hybrids. Previous genotyping methods (TaqMan assays, Sanger sequencing, etc.) are time consuming and heavily biased toward the detection of known variants and struggle with SV detection and interpretation^6^. Advancements in next-generation sequencing, particularly whole genome sequencing (WGS), with a rapid decrease in sequencing cost, as low as $100 per genome^7^, provide deeper insights into genetic and PGx variation. Computational tools are increasingly becoming available to interpret PGx variation and their functional implications^8^. However, challenges persist due to non-linearity in sequenced reads, resulting from the high sequence homology among functional genes and nonfunctional pseudogenes, which adversely affects sequence alignment^9,10^.

Previous studies on population-level PGx have enriched our comprehension of genetic variability and underscored the significance of considering varying frequencies across diverse populations^11–14^. However, these studies have often concentrated on only a few genes or a single gene, such as cytochrome P450 enzymes (CYPs)^15^ like *CYP2D6*, renowned for its high polymorphism, complex SVs, inclusion of a pseudogene (*CYP2D7*), and pivotal involvement in drug metabolism^16^. Another limitation of past research lies in their underpowered analyses due to smaller and less diverse sample sets. For instance, the Dutch Pharmacogenetics Working Group utilized WGS data to identify SVs within *CYP2D6* yet with a relatively small sample size (N = 547)^17^. Moreover, there are limited studies that have examined the role of SVs on pharmacogenes^18^. There are also limited computational methods for examining SVs within pharmacogenes^19^. Consequently, further endeavors are imperative to explore the impact of SVs on pharmacogenes and to enhance population-level PGx profiles.

In this study, we extended the work of PyPGx^20^, a Python package that can predict PGx genotypes and phenotypes from next-generation sequencing data by detecting SVs using a machine learning-based approach. We then used PyPGx to characterize PGx variation and phenotype landscapes at the population level, analyzing data from 2504 high-coverage WGS samples obtained from the 1000 Genomes Project (1KGP) encompassing five biogeographical populations: African (AFR), American (AMR), East Asian (EAS), European (EUR), and South Asian (SAS). 1KGP is an international effort to characterize global human genetic variation to improve our understanding of genetic contributors to human health and disease^21,22^. Notably, 1KGP relies on short-read sequencing, which raises challenges to identifying breakpoints and relative orientation of SVs^23^; long-read sequencing would be needed to confirm sequence of pharmacogenes. PyPGx does not identify breakpoints, but with its machine learning-based approach estimates copy number and the copy number signal helps to detect SVs. This study stands as a valuable resource, providing updated and precise population-level haplotype and phenotype information, while incorporating SVs.

## Materials and methods

### High-coverage WGS data

We downloaded publicly available, high coverage WGS data (mean = 30x) for unrelated, healthy 1KGP samples (N = 2504) from Phase III generated by the New York Genome Center^24^. There are 26 subpopulations that are grouped into five global populations (Supplementary Table 1). In accordance with recommendations^25^, we continue to use these five global populations, which have been grouped by greater genetic similarity in previous work^26^. We will refer to the populations as “1KGP” and then the referenced population. Briefly, we obtained FASTQ files from the European Nucleotide Archive (study accession: PRJEB31736) using the SRA Toolkit (v3.0.0; https://github.com/ncbi/sra-tools) to include only sequence reads that had been aligned to PGx regions of interest. Next, we re-aligned those reads to the Genome Reference Consortium Human Build 37 (GRCh37) reference genome using the ‘ngs-fq2bam’ command from the fuc package^20^.

### Star allele identification

From the WGS data we inferred star alleles in 58 PGx genes (Table 1) using the PyPGx package (v0.16.0)^20^ whose algorithm follows a modified version of the Stargazer genotyping pipeline^27,28^. The PyPGx pipeline starts by statistically phasing observed small variants (i.e., SNVs and indels) into two haplotypes per individual, which are then matched to candidate star alleles by cross-referencing against the target gene’s haplotype translation table. When a given haplotype produces multiple candidate star alleles, PyPGx sorts them by priority to pick the final allele to report. The sorting is performed as follows (in decreasing priority): (1) allele function (e.g. ‘No Function’ > ‘Normal Function’), (2) number of core variants (e.g. three SNVs > one SNV), (3) number of core variants that impact protein coding (e.g. two missense variants > one missense variant plus one intron variant), and (4) reference allele status (e.g. non-reference allele with two SNVs > reference allele with two SNVs). By default, PyPGx uses the Beagle program^29^ for statistical phasing with the entire 1KGP haplotype panel^21^ as reference solution. Next, per-base copy number is computed from read depth data through intra-sample normalization using a control gene as anchor. SVs are then detected from copy number data using a pre-trained support vector machine (SVM)-based classifier (see “Structural Variant Detection”). This version of PyPGx (v0.16.0) supports genotyping of 59 genes in total; briefly, these genes were selected based on their drug metabolism/response role (CPIC, FDA), allelic variation catalogs (PharmVar, PharmGKB, DGV), genotyping reference materials (GeT-RM), and overlap with tools like PGRNseq and Stargazer^20^. All the genes are listed in Table 1, except for *GSTT1*, which was excluded from the analysis because it is located on an alternative contig (chr1_KI270762v1_alt) for the human genome build GRCh38. For training the SVM-based classifier, both GRCh37 and GRCh38 builds were required. PyPGx outputs copy number and allele fraction profiles to allow users to manually inspect the quality of SV calls. Finally, candidate star alleles and SV results are combined to inform the final diplotype assignment (e.g. CYP2D6*1/*2).

**Table 1:** Overview of genes examined.

No.	Gene	Function	Haplotypes	SV	Phenotype	No.	Gene	Function	Haplotype	SV	Phenotype
1	*ABCB1*	Disposition	2	N/A	No	30	*CYP19A1*	Metabolism	4	N/A	No
2	*ABCG2*	Disposition	2	N/A	Yes	31	*CYP26A1*	Metabolism	2	N/A	No
3	*CACNA1S*	Target	1	N/A	Yes	32	*DPYD*	Excretion	42	N/A	Yes
4	*CFTR*	Target	7	N/A	Yes	33	*F5*	Other	2	N/A	Yes
5	*CYP1A1*	Metabolism	10	N/A	No	34	*G6PD*	Disease	24	N/A	No
6	*CYP1A2*	Metabolism	7	N/A	No	35	*GSTM1*	Metabolism	5	9	No
7	*CYP1B1*	Metabolism	9	N/A	No	36	*GSTP1*	Metabolism	3	N/A	No
8	*CYP2A6*	Metabolism	26	22	No	37	*IFNL3*	Other	2	N/A	Yes
9	*CYP2A13*	Metabolism	6	N/A	No	38	*NAT1*	Metabolism	6	N/A	No
10	*CYP2B6*	Metabolism	27	7	Yes	39	*NAT2*	Metabolism	11	N/A	No
11	*CYP2C8*	Metabolism	13	N/A	No	40	*NUDT15*	Metabolism	10	N/A	Yes
12	*CYP2C9*	Metabolism	22	N/A	Yes	41	*POR*	Disease	8	N/A	No
13	*CYP2C19*	Metabolism	16	N/A	Yes	42	*PTGIS*	Other	7	N/A	No
14	*CYP2D6*	Metabolism	64	19	Yes	43	*RYR1*	Disease	3	N/A	Yes
15	*CYP2E1*	Metabolism	11	9	No	44	*SLC15A2*	Excretion	2	N/A	No
16	*CYP2F1*	Metabolism	6	N/A	No	45	*SLC22A2*	Excretion	10	6	No
17	*CYP2J2*	Metabolism	6	N/A	No	46	*SLCO1B1*	Absorption	22	N/A	Yes
18	*CYP2R1*	Metabolism	2	N/A	No	47	*SLCO1B3*	Absorption	2	N/A	No
19	*CYP2S1*	Metabolism	4	N/A	No	48	*SLCO2B1*	Absorption	3	N/A	No
20	*CYP2W1*	Metabolism	5	N/A	No	49	*SULT1A1*	Metabolism	8	10	No
21	*CYP3A4*	Metabolism	23	N/A	No	50	*TBXAS1*	Other	8	N/A	No
22	*CYP3A5*	Metabolism	5	N/A	Yes	51	*TPMT*	Metabolism	13	N/A	Yes
23	*CYP3A7*	Metabolism	2	N/A	No	52	*UGT1A1*	Excretion	6	N/A	Yes
24	*CYP3A43*	Metabolism	3	N/A	No	53	*UGT1A4*	Excretion	12	5	No
25	*CYP4A11*	Metabolism	3	N/A	No	54	*UGT2B7*	Excretion	3	N/A	No
26	*CYP4A22*	Metabolism	9	N/A	No	55	*UGT2B15*	Excretion	10	9	No
27	*CYP4B1*	Metabolism	5	N/A	No	56	*UGT2B17*	Excretion	5	8	No
28	*CYP4F2*	Metabolism	4	2	No	57	*VKORC1*	Target	2	N/A	No
29	*CYP17A1*	Metabolism	1	N/A	No	58	*XPC*	Other	2	N/A	No

Number: No.; Structural variant: SV.

To facilitate parallel computing, we divided the samples into ten non-overlapping batches of N = 250 except for the last one with N = 254. For every batch we then ran the ‘run-ngs-pipeline’ command from PyPGx for each target gene with three input files: (1) a multi-sample VCF file, (2) a depth of coverage file, and (3) a control statistics file. These input files were created for every batch of BAM files using the ‘create-input-vcf’, ‘prepare-depth-of-coverage’, and ‘compute-control-statistics’ commands from PyPGx, respectively. All of the genotyping analyses shown in the results section were conducted using the *VDR* gene as the control locus.

### Comparison of haplotype and phenotype frequencies

**Fig. 1:**
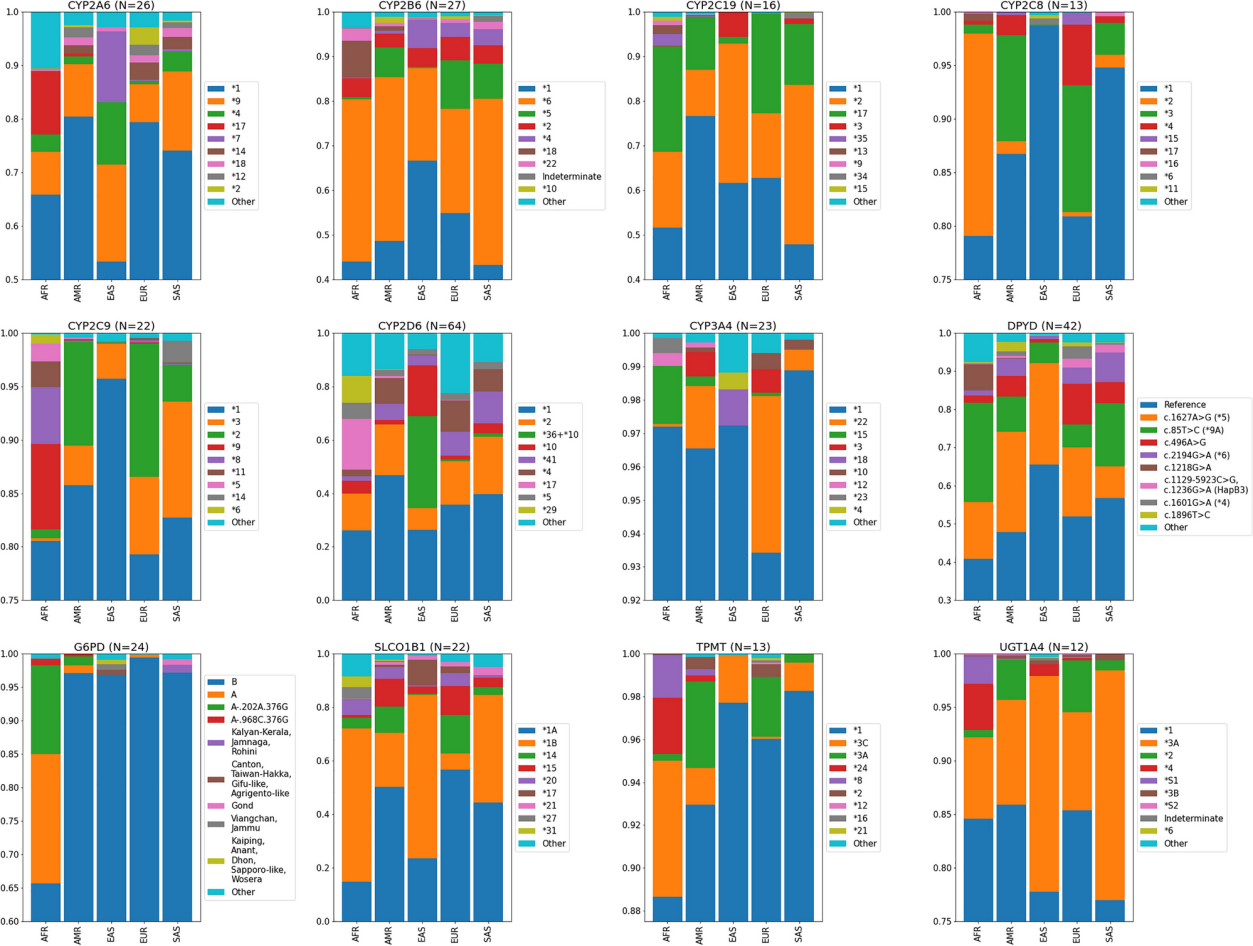
Star allele frequencies for the entire 1KGP dataset. The total number of unique alleles is indicated for each gene.

We compared our calculated frequencies of select genes across ancestral populations to the Pharmacogenomics Knowledge Base (PharmGKB)^30^, in which frequencies were estimated using the formula for Hardy Weinberg equilibrium based on reported allele frequencies. PharmGKB’s grouping system is based on genetic similarity using data from the 1KGP and the Human Genome Diversity Project^31^. Differences in the population grouping between 1KGP and PharmGKB can introduce biases, as PharmGKB has finer stratification and may limit comparisons. We compared our haplotype frequencies of 12 of the most polymorphic genes (Fig. 1) to haplotype frequencies within PharmGKB; seven of the twelve genes were available: *CYP2B6*, *CYP2C9, CYP2C19*, *CYP2D6*, *DPYD*, *G6PD*, and *TPMT*. Ratios (our values over the literature) were calculated, and values that differed by at least a twofold difference in either direction were highlighted. PharmGKB has nine biogeographical groups within the dataset^31^. 1KGP-AFR populations were compared to PharmGKB-Sub-Saharan African (SSA); 1KGP-AMR was compared to PharmGKB-American (AME) when available; 1KGP-EAS was compared to PharmGKB-East Asian (EAS); 1KGP-EUR was compared to PharmGKB-European (EUR) populations; 1KGP-SAS was compared to PharmGKB-Central/South Asian (SAS). PyPGx produces phenotypes for 16 genes (see “Phenotype Prediction”). As above, we compared 1KGP phenotype frequencies to PharmGKB; ten of the sixteen genes were available: *ABCG2, CYP2B6*, *CYP2C9*, *CYP2C19*, *CYP2D6*, *CYP3A5*, *DPYD, NUDT15, TPMT* and *UGT1A1*. For both haplotype and phenotype comparisons, *SLCO1B1* was excluded because PharmGKB and PyPGx used different PGx databases. PharmGKB provided only activity scores for *CYP2D6* and *DPYD*, and translation tables from PyPGx were utilized to convert these activity scores into phenotype predictions.

### Structural variation detection

PyPGx supports SV detection in 11 of the 58 genes assessed. These include *CYP2A6*, *CYP2B6*, *CYP2D6*, *CYP2E1*, *CYP4F2*, *GSTM1*, *SLC22A2*, *SULT1A1*, *UGT1A4*, *UGT2B15*, and *UGT2B17*. SVs are detected from per-base copy number data using a pre-trained SVM-based multiclass classifier using the one-vs-rest strategy. This means the classifier cannot detect SVs that it has not seen before. Therefore, for each gene with SV we manually combed through individual samples and their copy number and allele fraction profiles to generate training and testing datasets for both known and novel SVs. For rare SVs we sought to synthetically increase sample size through simulation using the ‘pypgx.sdk.utils.simulate_copy_number’ method which introduces random noise to existing samples. Once datasets were gathered, we combined them with existing datasets from PyPGx and then re-trained SVM classifiers for both GRCh37 and GRCh38.

The final training and testing datasets and corresponding classification accuracy are summarized in Supplementary Table 2 and can be accessed from https://github.com/sbslee/pypgx-data. After the update, the sample size increased on average three-fold in the training dataset (from N = 93.0 to N = 279.7) and two-fold in the testing dataset (from N = 29.0 to N = 67.1). Similarly, the average number of unique SVs increased from 4.1 to 9.6 for the train set and from 3.6 to 9.6 for the test set, significantly increasing the complexity of the SV space. The training accuracy was 100% for all of the genes except for *CYP2D6* and *SULT1A1* which still showed a high accuracy ranging from 0.990 to 0.997 depending on the reference genome build. The testing accuracy was 100% for all of the genes. Of note, PyPGx also supports SV detection in the *G6PD* gene, but it is solely for sex determination because the gene is located on the X chromosome. Since there was no need for additional training, the SVM classifier for *G6PD* was not updated in this study. To determine if our novel SVs overlapped with previously characterized SVs, we compared estimated endpoints from the copy number profile produced by PyPGx to the UCSC Genome Browser on Human (GRCh37/hg19) (https://genome.ucsc.edu/), including the Database of Genomic Variants^32–34^.

### Phenotype prediction

In addition to diplotype calls, the ‘run-ngs-pipeline’ command from PyPGx automatically produces predicted phenotypes if the target gene is one of the 16 genes with a genotype–phenotype table from CPIC. Nine genes (*CYP2B6*, *CYP2C19*, *CYP2C9*, *CYP2D6*, *CYP3A5*, *DPYD*, *NUDT15*, *TPMT*, *UGT1A1*) produce prediction of drug metabolizer status ranging from poor to ultrarapid metabolizer^35^; *CFTR*, *F5*, and *IFNL3* produce prediction of responder status to certain therapeutics (i.e. favorable vs. unfavorable response)^36–38^; *ABCG2* and *SLCO1B1* produce prediction of enzymatic function status ranging from poor to increased function^39^; *CACNA1S* and *RYR1* produce prediction of malignant hyperthermia susceptibility^40^.

More specifically, there are two phenotype prediction methods in PyPGx. The first method uses a simple diplotype-to-phenotype mapping system provided by CPIC. For instance, CYP2B6*1/*29 and *6/*6 diplotypes will be assigned an intermediate metabolizer and a poor metabolizer, respectively. The second method uses a standard unit of enzyme activity known as an activity score^41^. For example, the fully functional reference CYP2C9*1 allele is assigned a value of 1, decreased-function alleles such as CYP2C9*2 and *5 receive a value of 0.5, and nonfunctional alleles including CYP2C9*3 and *6 have a value of 0. The sum of values assigned to both alleles constitutes the activity score of a diplotype. Consequently, subjects with CYP2C9*1/*1, *1/*2, and *3/*6 diplotypes have an activity score of 2 (normal metabolizer), 1 (intermediate metabolizer), and 0 (poor metabolizer), respectively. PyPGx uses the second method for the *CYP2C9*, *CYP2D6*, and *DPYD* genes and the first method for the rest of the genes. The burden of “abnormal, priority, and high risk” responses according to CPIC (Supplementary Table 3) for each individual and ancestral population was determined by counting the number of genes with these predicted responses (see “Supplementary Materials and Methods”). From the WGS data we identified a large number of SNVs and indels that were not used to define star alleles. To explore the potential contribution of these variants to enzymatic activity, SNVs and indels were functionally annotated using the Combined Annotation Dependent Depletion (CADD) tool^42^ (Supplementary Table 4) and were uploaded into the web interface, Ensembl Variant Effect Predictor (VEP) (Assembly: GRCh37.p13)^43,44^ (see “Supplementary Materials and Methods”).

## Results

### Haplotype variation patterns

We applied the PyPGx program to the high coverage WGS data from 1KGP to generate diplotype calls in 58 pharmacogenes from 2,504 unrelated samples. All individual PyPGx calls (N = 145,232), including diplotypes, SVs, and predicted phenotypes, can be found in Supplementary Table 5. Of those calls, we observed a total of 538 unique star alleles, including reference alleles (Table1; Table 2). The *CYP2D6* gene had the highest number of unique alleles, 64, followed by *DPYD* with 42, *CYP2B6* with 27, *CYP2A6* with 26, and *G6PD* with 24. Conversely, the *CACNA1S* and *CYP17A1* genes showed the least polymorphism with zero non-reference alleles. We then computed star allele frequencies for each gene for each of the five global populations in 1KGP (Supplementary Table 6). Figure 1 shows the relative proportion of observed star alleles for the 12 most polymorphic genes that have the highest number of unique alleles.

**Table 2:** List of alleles.

Gene	Reference	Alleles
*ABCB1*	*1	*2
*ABCG2*	Reference	rs2231142
*CACNA1S*	Reference	None
*CFTR*	Reference	R74W; S549N; D1270N; F1074L; R1070W; F508del
*CYP17A1*	Reference	None
*CYP19A1*	*1	*2; *3; *4
*CYP1A1*	*1	*2A; *2B; *2C; *4; *5; *10; *11; *12; *13
*CYP1A2*	*1A	*1C; *1F; *1 K; *1L; *6; *20
*CYP1B1*	*1	*2; *3; *4; *5; *6; *7; *22; *23
*CYP26A1*	*1	*2
*CYP2A13*	*1	*2; *3; *6; *7; *8
*CYP2A6*	*1	*1 × 2 (dup); *2; *4 (del); *7; *9; *9 × 2 (dup); *11; *12 (hyb); *13; *14; *15; *17; *18; *19; *20; *21; *23; *24; *25; *26; *28; *31; *34 (hyb); *39; Indeterminate
*CYP2B6*	*1	*1 × 2 (dup); *2; *3; *4; *5; *6; *7; *9; *10; *11; *12; *13; *15; *17; *18; *19; *20; *22; *22 × 2 (dup); *23; *24; *26; *27; *29 (hyb); *35; Indeterminate
*CYP2C19*	*1	*2; *3; *4; *8; *9; *10; *13; *15; *17; *22; *24; *30; *34; *35; *39
*CYP2C8*	*1	*2; *3; *4; *5; *6; *11; *12; *14; *15; *16; *17; *18
*CYP2C9*	*1	*2; *3; *5; *6; *7; *8; *9; *11; *12; *13; *14; *16; *29; *31; *33; *44; *45; *61; *63; *66; *68
*CYP2D6*	*1	*1 × 2 (dup); *1 × 3 (multip); *2; *2 × 2 (dup); *2 × 3 (multip); *3; *4; *4 × 2 (dup); *4 × 3 (multip); *5 (del); *6; *7; *9; *9 × 2 (dup); *10; *10 × 2 (dup); *11; *13 + *1 (hyb); *14; *15; *17; *17 × 2 (dup); *20; *21; *22; *28; *29; *29 × 2 (dup); *31; *35; *35 × 2 (dup); *36 + *10 (hyb); *36 × 2 + *10 (hyb); *36 × 3 + *10 (hyb); *40; *41; *42; *43; *43 × 2 (dup); *45; *45 × 2 (dup); *46; *53; *56; *59; *68 + *4 (hyb); *68 × 2 + *4 (hyb); *71; *74; *82; *86; *88; *99; *106; *108; *111; *112; *113; *117; *121; *125; *136; Indeterminate
*CYP2E1*	*1	*1 × 2 (dup); *2; *3; *4; *5; *7; *7 × 2 (dup); *7 × 3 (multip); *S1 (del); Indeterminate
*CYP2F1*	*1	*2; *3; *4; *5; *6
*CYP2J2*	*1	*4; *5; *7; *8; *9
*CYP2R1*	*1	*2
*CYP2S1*	*1	*2; *3; *4
*CYP2W1*	*1	*2; *3; *4; *6
*CYP3A4*	*1	*2; *3; *4; *5; *6; *7; *8; *9; *10; *11; *12; *15; *16; *18; *19; *20; *21; *22; *23; *24; *28; *35
*CYP3A43*	*1	*2; *3
*CYP3A5*	*1	*3; *4; *6; *7
*CYP3A7*	*1	*2
*CYP4A11*	*1	F434S; S353G
*CYP4A22*	*1	*3; *4; *5; *8; *9; *12; *13; *15
*CYP4B1*	*1	*2; *3; *4; *5
*CYP4F2*	*1	*2; *3; *DEL (del)
*DPYD*	Reference	c.61C > T; c.85 T > C (*9A); c.451A > G; c.496A > G; c.525G > A; c.557A > G; c.775A > G; c.868A > G; c.967G > A; c.1024G > A; c.1108A > G; c.1181G > T; c.1218G > A; c.1260 T > A; c.1314 T > G; c.1349C > T; c.1358C > G; c.1371C > T; c.1577C > G; c.1601G > A (*4); c.1615G > A; c.1627A > G (*5); c.1679 T > G (*13); c.1774C > T; c.1896 T > C; c.1905C > G; c.2161G > A; c.2194G > A (*6); c.2195 T > G; c.2279C > T; c.2303C > A; c.2336C > A; c.2582A > G; c.2657G > A (*9B); c.2846A > T; c.2915A > G; c.2978 T > G; c.3067C > A; c.1905 + 1G > A (*2A); c.295_298delTCAT (*7); c.1129-5923C > G, c.1236G > A (HapB3)
*F5*	Reference	Leiden
*G6PD*	B	A; Gond; Asahi; Aures; Ilesha; Orissa; Sibari; Chinese-5; Ube Konan; Palestrina; Mira d’Aire; A-.202A.376G; A-.968C.376G; Sierra Leone; Coimbra Shunde; Viangchan,Jammu; Chinese-4,Quing Yan; Union,Maewo,Chinese-2,Kalo; Kalyan-Kerala,Jamnaga,Rohini; Kaiping,Anant,Dhon,Sapporo-like,Wosera; Seattle,Lodi,Modena,Ferrara-II,Athens-like; Canton,Taiwan-Hakka,Gifu-like,Agrigento-like; Mediterranean,Dallas,Panama Sassari,Cagliari,Birmingham
*GSTM1*	*A	*0 (del); *B; *Ax2 (dup); Indeterminate
*GSTP1*	*A	*B; *C
*IFNL3*	Reference	rs12980275
*NAT1*	*4	*11; *14; *15; *17; *22
*NAT2*	*4	*5; *6; *7; *10; *12; *13; *14; *22; *23; *24
*NUDT15*	*1	*2; *3; *4; *5; *6; *9; *13; *14; *15
*POR*	*1	*2; *11; *28; *36; *37; *42; *45
*PTGIS*	*1	*1D; *1E; *1G; *1 J; *3; *5
*RYR1*	Reference	c.1654C > T; c.1840C > T
*SLC15A2*	*1	*2
*SLC22A2*	*1	*2; *3; *4; *6; *7; *S1 (del); *S2 (del); *K432Q; Indeterminate
*SLCO1B1*	*1A	*1B; *5; *9; *14; *15; *16; *17; *19; *20; *21; *22; *24; *27; *28; *29; *30; *31; *32; *35; *S1; *S2
*SLCO1B3*	Reference	rs7311358
*SLCO2B1*	*1	*S1; *S464F
*SULT1A1*	*1	*1 × 2 (dup); *1 × 3 (multip); *2; *2 × 2 (dup); *2 × 3 (miltip); *DEL (del); Indeterminate
*TBXAS1*	*1	*2; *3; *5; *6; *7; *8; *9
*TPMT*	*1	*2; *3A; *3C; *6; *8; *12; *16; *21; *24; *32; *33; *40
*UGT1A1*	*1	*6; *28; *36; *80 + *28; *80 + *37
*UGT1A4*	*1	*2; *3A; *3B; *4; *6; *7; *8; *S1 (del); *S2 (del); *S3 (dup); Indeterminate
*UGT2B15*	*1	*2; *4; *5; *6; *S1 (del); *S2 (del); *S3 (del); *S4 (del); Indeterminate
*UGT2B17*	*1	*2 (del); *S1 (del); *S2 (del); *S3 (del)
*UGT2B7*	*1	*2; *3
*VKORC1*	Reference	rs9923231
*XPC*	Reference	rs2228001

Structural variant-defined alleles are indicated by “del” (deletion), “dup” (duplication), “multip” (multiplication), and “hyb” (hybrid).

Among the 12 most polymorphic genes, 7 had population-specific allele frequencies available from PharmGKB for comparison (Supplementary Table 7). After comparing 208 unique star alleles across the seven genes, 97 unique alleles overlapped with previously reported PharmGKB frequencies. Over half of the alleles were consistent with the literature and fell within a twofold range (59.6%; N = 58/97). To illustrate, the frequencies for both CYP2D6*17, a reduced-function haplotype that is common in individuals of African ancestry (e.g., c.1023C > T; rs28371706)^45,46^, and CYP2C19*2, a common loss-of-function haplotype (e.g., c.681G > A; rs4244285)^47^, were consistent with the previously reported frequencies across populations. For example, the frequency for CYP2D6*17 for both 1KGP-AFR and PharmGKB-SSA was around 19%, and for the rest of the populations, both 1KGP and PharmGKB frequencies were ≤ 1%. For CYP2C19*2 all compared populations had similar frequencies: 1KGP-AFR (17%) and PharmGKB-SSA (16%); 1KGP-EAS (31%) and PharmGKB-EAS (28%); 1KGP-EUR (15%) and PharmGKB-EUR (15%); 1KGP-SAS (36%) and PharmGKB-SAS (27%); 1KGP-AMR (10%) and PharmGKB-AME (12%) (Supplementary Table 7).

Although the majority of our frequency calls fell within a two-fold range, our analysis revealed that 40.2% of haplotype calls (N = 39/97) exhibited a difference of two-fold or greater. Among these, 30.3% (N = 30/97) showed fold-changes ranging from 2x to 10x. Notably, there were “extreme” cases, accounting for 10.1% (N = 10/97), where fold-changes exceeded 10x. Half of these extreme cases (N = 5) ranged from approximately 15x to 160x, yet all had frequencies below 0.6% in both 1KGP and PharmGKB databases, notably involving *G6PD Ube Konan* (EAS), *DPYD c.1905C* > *G* (EUR), *DPYD c.967G* > *A* (EUR), CYP2C19*4 (AMR), and CYP2D6*3 (AMR). Consequently, these extreme cases lack significant clinical relevance. In PharmGKB, a wide range of reported frequencies resulted in additional extreme cases with fold-changes ranging from approximately 15x to 70x  (N = 3). Notable instances include PharmGKB-SSA involving CYP2B6*11 (range: 0–13%; average 7.1%) and *15 (range: 0–7.7%; average: 1.5%), as well as PharmGKB-AMR, featuring CYP2C9*8 (range: 0–4.4%). For all three cases, a frequency of 0.1% in 1KGP was determined, aligning with studies included in PharmGKB and other databases such as dbSNP. It is important to note that PharmGKB-AMR and SSA represent heterogeneous groups, with sub-populations potentially exhibiting varying frequencies. Furthermore, we observed extreme cases (N = 2) where the *DPYD c.1896T* > *C* haplotype (rs17376848)^48^, resulting in normal enzyme function, exhibited a 125x fold-difference for EAS and an 18x fold-difference for SAS. Given that both the reference allele (rs1801265 in GRCh37) and the *c.1896 T* > *C* allele share: (1) normal function, (2) the same number of core variants (N = 1), the deciding factor when PyPGx was choosing which allele to report, if both rs1801265 and rs17376848 were present in the same haplotype, was the impact on protein coding (refer to "Star allele identification" for more details). Notably, *DPYD* reference harbors a missense variant, while *c.1896T* > *C* carries a synonymous variant, leading PyPGx to prioritize the reference over *c.1896T* > *C*.

### Detection of known and novel SVs

**Fig. 2:**
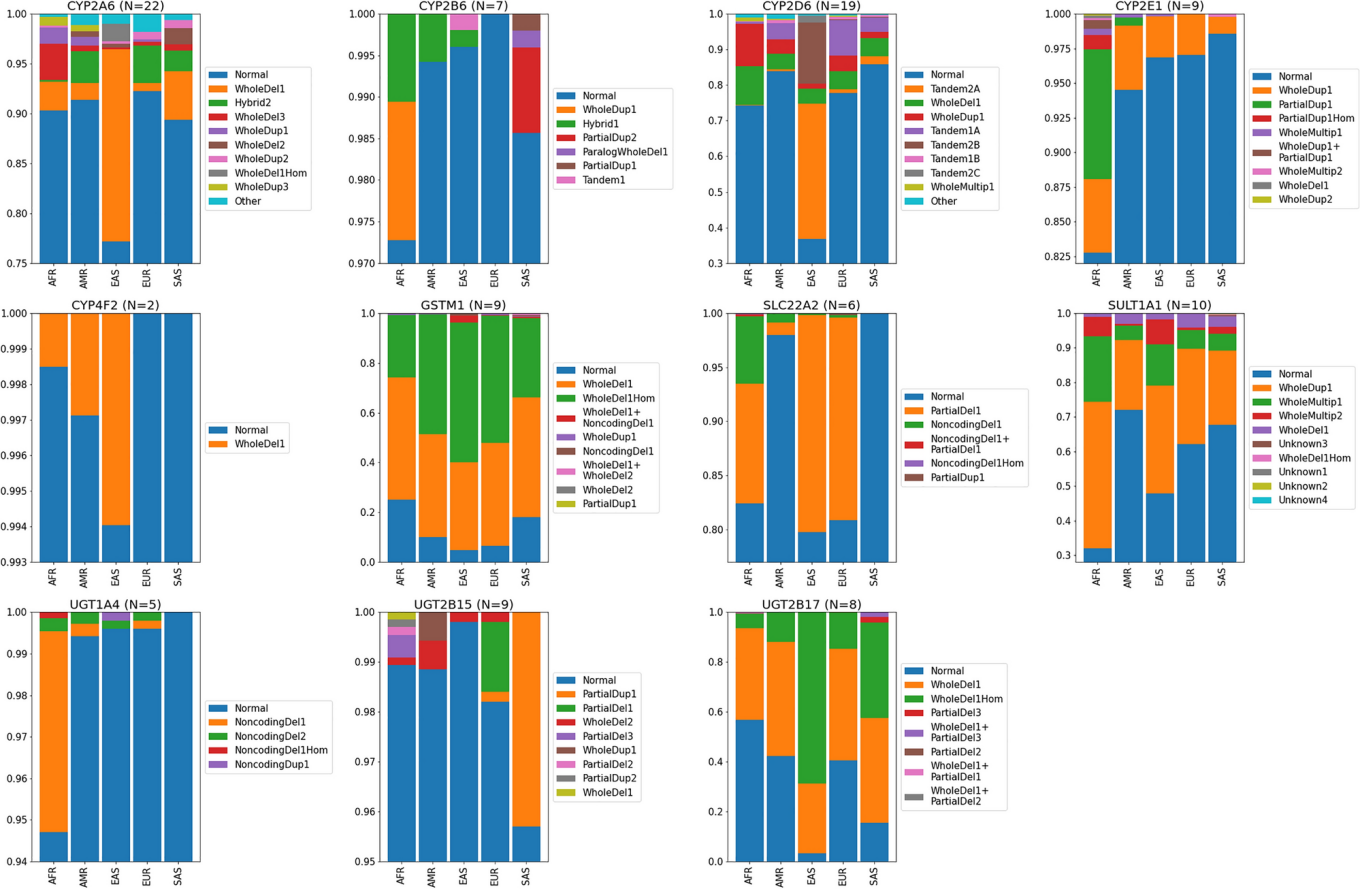
Structural variant frequencies for the entire 1KGP dataset. The total number of unique variants is indicated for each gene.

We identified 53 known and novel SV-carrying star alleles in the 11 genes that were assessed for the presence of SV (Table 2). The alleles consisted of gene deletions (e.g., CYP2A6*4, CYP2D6*5, GSTM1*0), duplications (e.g., CYP2A6*1 × *2*, CYP2E1*7 × *2*, GSTM1*Ax2), multiplications (e.g., CYP2D6*36 × *3* + *10, CYP2E1*7 × *3*, SULT1A1*2 × *3*), and hybrids (e.g., CYP2A6*12, CYP2B6*29, CYP2D6*68 + *4). These were collectively found in 22.9% (6,305/27,544) of the diplotypes examined for SV. The relative proportion of detected SVs for the 11 genes is shown in Fig. 2. About 1.2% (329/27,544) of the diplotypes were returned as ‘Indeterminate’ due to the difficulty in interpretation of SV.

Among the SVs identified, one stood out for its high frequency (34.4%) among 1KGP-EAS: CYP2D6*36 + *10 (hybrid), which, notably, was not cataloged in PharmGKB for comparison. However, a comprehensive literature review spanning PubMed publications from 1995 to 2015 revealed an average allele frequency of 26.41% (range 22.45–32.65%)^49^ for CYP2D6*36 + *10 among populations of East Asian descent. In contrast, the average reported frequency for CYP2D6*10 in East Asian populations was 42.58% (range: 8.6–64.1%)^49^, while our estimation yielded a frequency of 18.8%. This variance suggests challenges in accurately determining the frequency of the CYP2D6*36 + *10. Moreover, our analysis allowed us to ascertain the frequencies of CYP2D6*36 × *2* + *10 and *36 × *3* + *10 across various populations (Supplementary Table 6). It is worth noting that both PharmGKB and the extensive literature review rely on available studies to estimate SV frequencies. However, these studies often employ genotyping assays prone to error and influenced by the diverse methodologies of different laboratories. In contrast, our WGS approach offers a more direct and updated method to determine frequencies for haplotypes containing SVs.

**Fig. 3:**
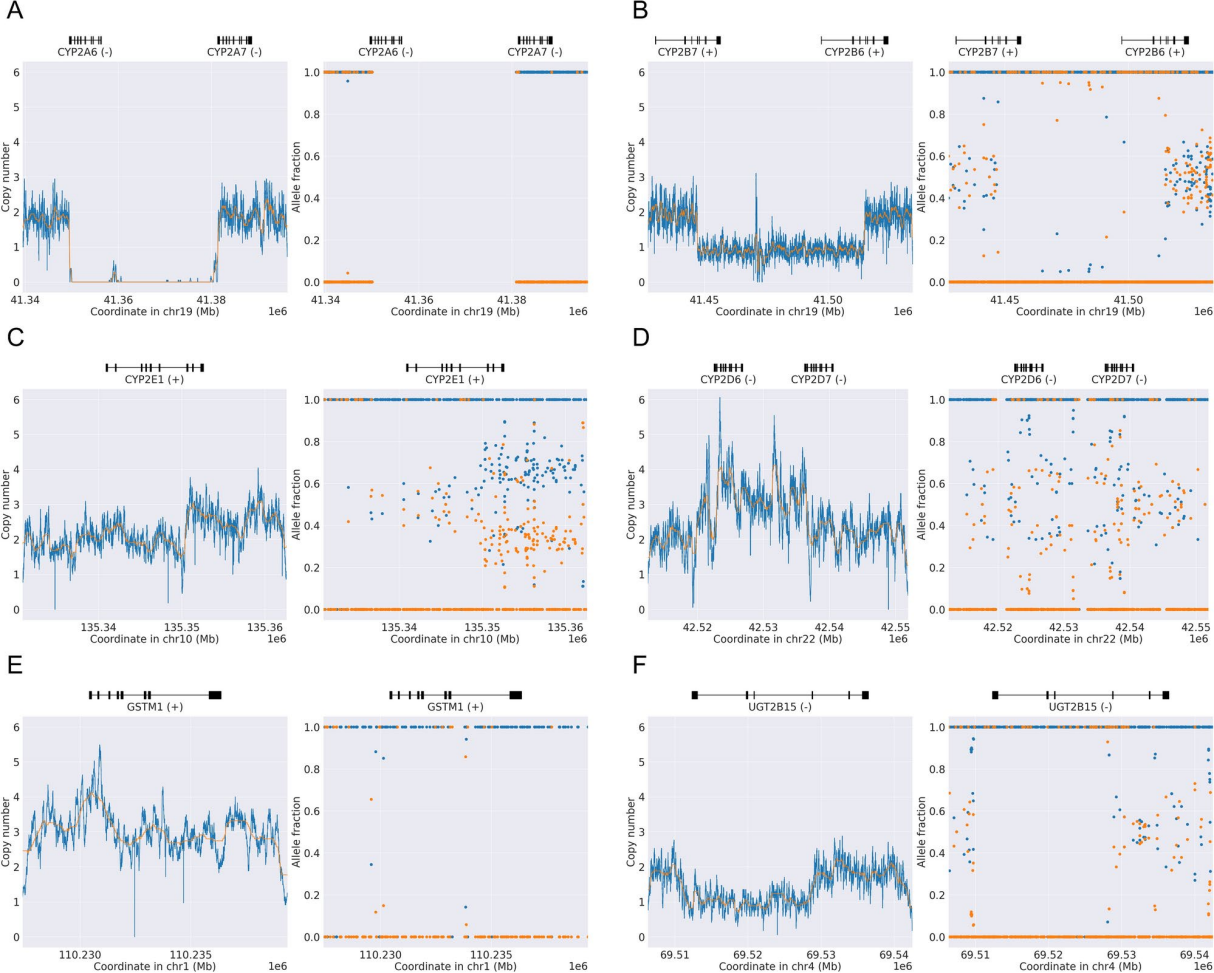
Examples of known structural variants in pharmacogenes detected by PyPGx. Each panel contains a copy number profile and an allele fraction profile produced by PyPGx. (**A**) Homozygous gene deletion in East Asian sample NA18952 with a CYP2A6*4/*4 diplotype. (**B**) *CYP2B7/CYP2B6* hybrid in African sample NA18871 with a CYP2B6*18/*29 diplotype. (**C**) Partial gene duplication in African sample NA18511 with a CYP2E1*1/*S1 diplotype. (**D**) *CYP2D6/CYP2D7* hybrid in African sample NA18947 with a CYP2D6*1/*36 + *10 diplotype. (**E**) Whole gene duplication in European sample NA11894 a GSTM1*A/*Ax2 diplotype. (**F**) Partial gene deletion in European sample NA11931 with a UGT2B15*1/*S1 diplotype.

**Fig. 4:**
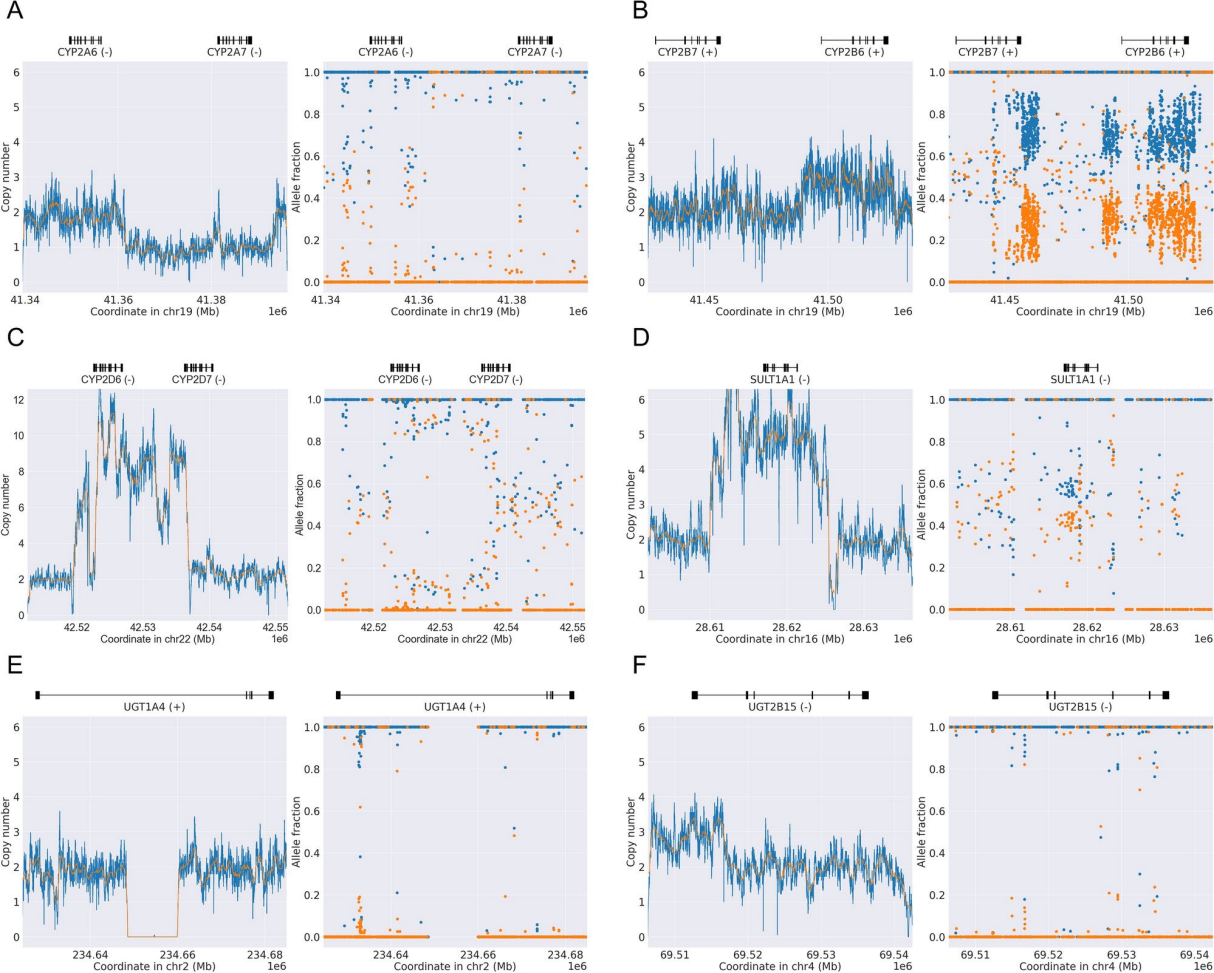
Examples of novel structural variants in pharmacogenes detected by PyPGx. Each panel contains a copy number profile and an allele fraction profile produced by PyPGx. (**A**) Whole gene deletion of *CYP2A7*, which is a pseudogene of *CYP2A6*, in East Asian sample HG00625 with a CYP2A6*1/*7 diplotype. (**B**) Whole gene duplication in African sample NA19190 with CYP2B6*6/*22 × *2* diplotype. (**C**) Complex *CYP2D6/CYP2D7* hybrid variation in East Asian HG00458 with an indeterminate diplotype. (**D**) Whole gene multiplication of *SULT1A1* in African sample NA19143 with an indeterminate diplotype. (**E**) Homozygous partial gene deletion in African sample HG03479 with a UGT1A4*S1/*S1 diplotype. (F) Partial gene duplication of *UGT2B15* in African sample HG03082 with an indeterminate diplotype.

Figure 3 showcases six representative examples of well-known SVs, demonstrating PyPGx’s robust detection capabilities. Additionally, Fig. 4 presents six representative examples of novel SVs identified in this study. We categorize these predicted SVs as ‘novel’ because they have not been previously utilized to define star alleles. However, it is likely that some of these novel SVs have been identified by others, not necessarily within the context of pharmacogenomics, given the extensive study of the 1KGP dataset. Therefore, to identify previously reported SVs within the novel SV gene regions, we utilized the UCSC Genome Browser. One representative example is illustrated in Supplementary Fig. 1. Among our findings, we identified gssvL58571, a copy number variation region (chr19:41,352,371–41,397,661), as a potential candidate for a whole-gene deletion of *CYP2A7* (Fig. 4A). Additionally, nssv3567503 represents a gain of CNV region (chr19:41,497,274–41,558,271), indicating a whole-gene duplication of *CYP2B6* (Fig. 4B). Interestingly, this duplication, CYP2B6*22 × *2*, was previously detected by PyPGx in an Vindija Neanderthal individual^50^. Another notable finding is esv33893, presenting a gain and loss variation (chr22:42,522,622–42,538,228), suggestive of multiple copies of a complex *CYP2D6/CYP2D7* hybrid variation (Fig. 4C). Furthermore, we highlight gssvG14505, delineating a CNV region (chr16:28,609,490–28,626,916), which included the same sample (NA19143) utilized in our study, indicating a whole-gene multiplication in *SULT1A1* (Fig. 4D). Lastly, esv25622 represents a loss variation (chr2:234,648,159–234,659,534), one of the few candidates showing a homozygous partial gene deletion in *UGT1A4* (Fig. 4E), while gssvG27730 represents a gain of CNV region (chr4:69,217,756–69,592,846), overlapping with a partial gene duplication in *UGT2B15* (Fig. 4F).

### PGx phenotype frequencies

**Fig. 5:**
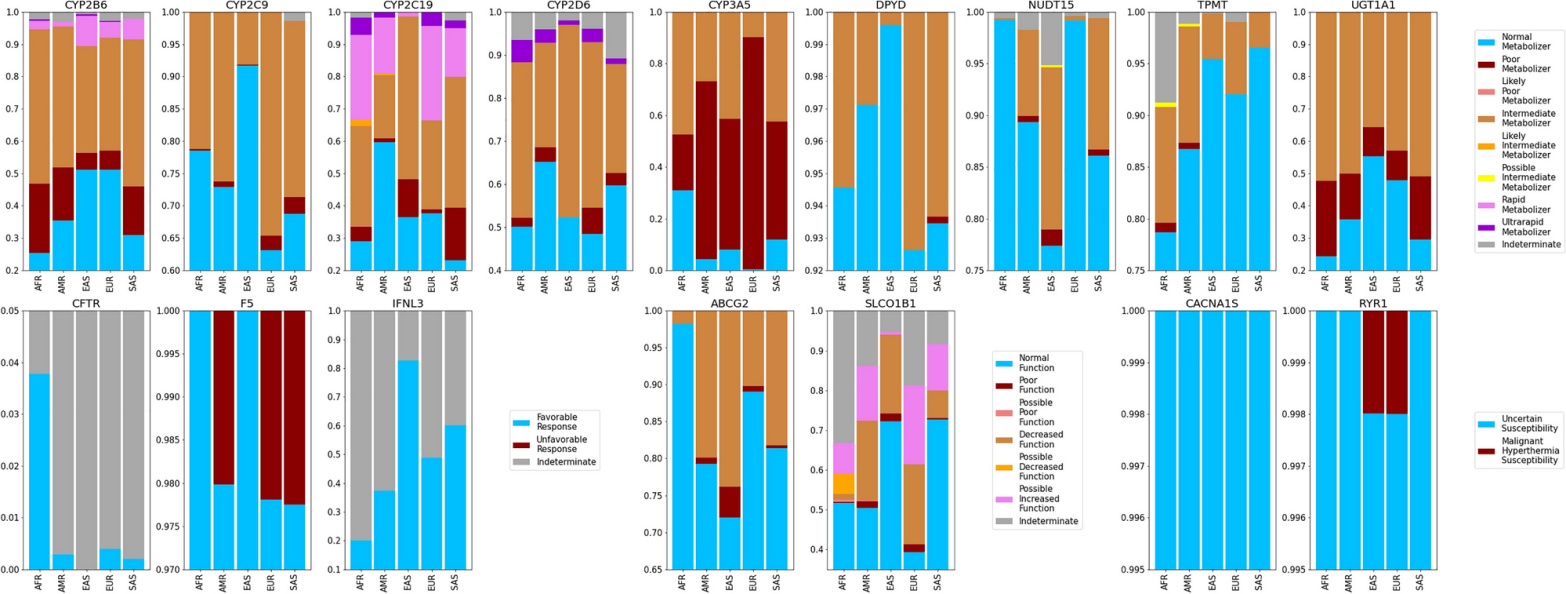
Phenotype frequencies for the entire 1KGP dataset. Upper panels show the nine genes associated with drug metabolizer status. The *CFTR*, *F5*, and *IFNL3* genes produce prediction of responder status to certain therapeutics. Two genes (*ABCG2* and *SLCO1B1*) produce prediction of enzymatic function status. The *CACNA1S* and *RYR1* genes produce prediction of malignant hyperthermia susceptibility.

**Fig. 6:**
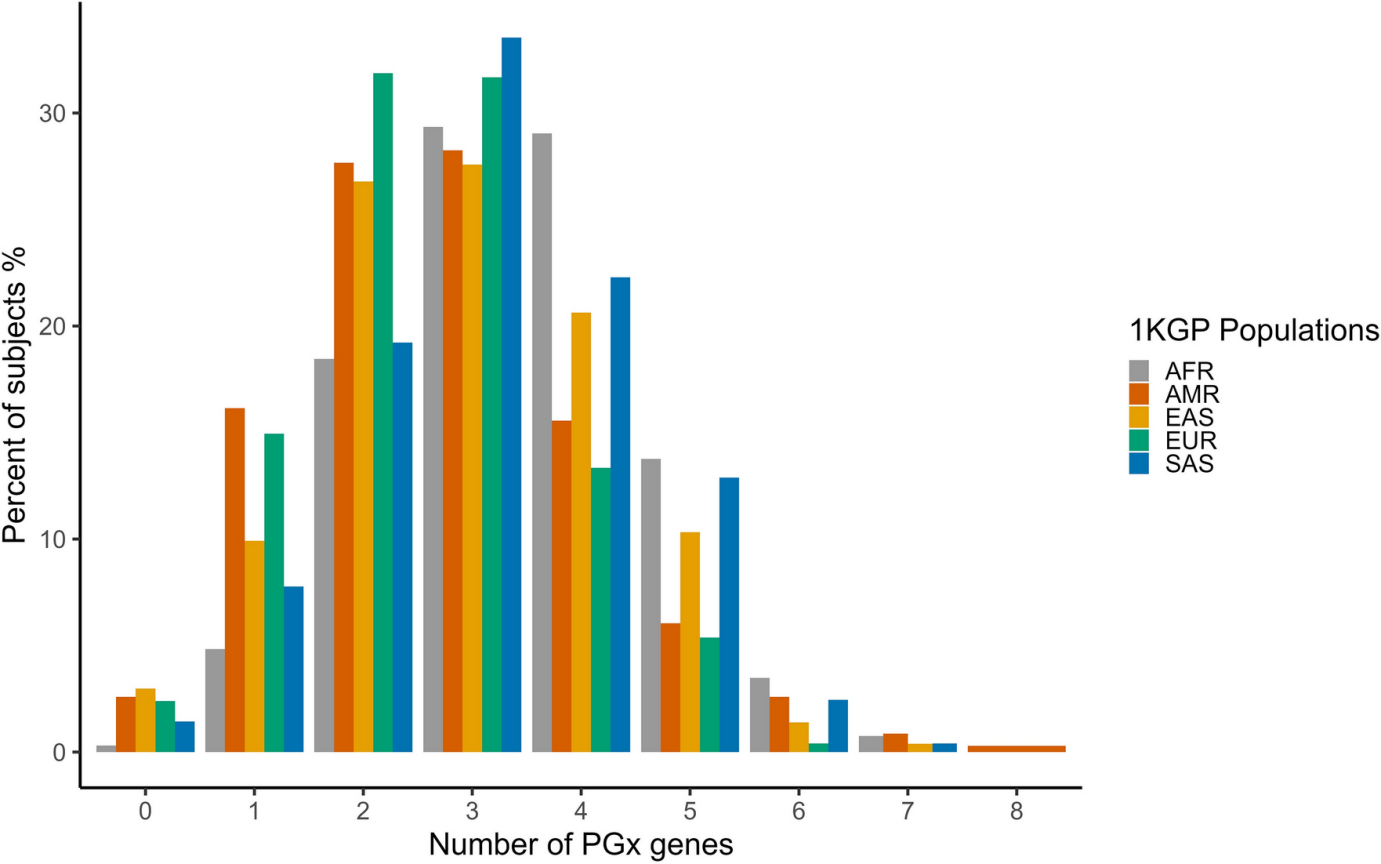
Number of PGx genes with atypical drug response as determined by CPIC. Distribution of pharmacogenes with a predicted non-typical response across the entire 1KGP dataset.

PyPGx generated predicted phenotypes utilizing genotype–phenotype translation tables from CPIC across 16 genes. The frequencies of these phenotypes are outlined in detail in Supplementary Table 8 and summarized in Fig. 5. Furthermore, we assessed the prevalence of gene-phenotype patterns across thirteen genes, leading to abnormal, priority, and high-risk phenotypes according to CPIC guidelines (Supplementary Table 3; Fig. 6). On average, subjects exhibited approximately 3 non-typical response phenotypes for the thirteen pharmacogenes, with 98.2% of individuals demonstrating at least one non-typical drug response. For example, one individual from 1KGP-AFR exhibited the following predicted phenotypes: *CYP2B6*-poor metabolizer, *CYP2C19*-rapid metabolizer, and *CYP2D6*-intermediate metabolizer.

Additionally, we compared phenotype frequencies to those in PharmGKB for 10 out of the 16 genes (Supplementary Table 9). Among 88 unique gene-phenotype pairs (e.g., *CYP2D6*-Normal Metabolizer), 59.0% (N = 52/88) overlapped within the PharmGKB database. Notably, 20 of these pairs exhibited at least a twofold difference in frequency across populations. Among these, 2 showed fold-changes greater than 10x, and 18 showed fold-changes between 2x to 10x. To investigate the identified differences in phenotype frequencies, we examined the most significant disparities in DPYD-poor metabolizer (SAS) and DPYD-intermediate metabolizer (AFR) (Supplementary Table 9). For DPYD-poor metabolizers, SAS exhibited a 53-fold difference, despite both 1KGP and PharmGKB reporting frequencies of less than 0.2%. Similarly, for DPYD-intermediate metabolizers, AFR/SSA displayed a fold-difference of 69.8x. However, within PharmGKB-SSA, there is a comparable frequency for DPYD-likely intermediate metabolizer to 1KGP-AFR DPYD-intermediate metabolizer; hence, “likely” phenotypes could impact our observed fold-differences.

### Clinically relevant PGx variants

From the WGS data we found a total of 190,398 unique small nucleotide variants in 58 genes, of which only 502 (0.26%) were used to define star alleles. To assess the potential clinical relevance of the remaining 189,910 variants not part of any known star allele nomenclature, we filtered the variants based on their annotated consequences (‘stop_gained’, ‘frameshift’, ‘missense’, ‘splice_donor’, and ‘regulatory’). This resulted in 210 variants (Supplementary Table 4). The distribution of these variants across populations was: 1KGP-AFR (N = 67) > 1KGP-SAS (N = 56) > 1KGP-EAS (N = 54) > 1KGP-EUR (N = 41) > 1KGP-AMR (N = 27). The consequences for the 210 variants across 48 genes are shown in Supplementary Fig. 2. We identified eight unique SNVs previously reported to have ClinVar significance: ‘Likely pathogenic’ in *RYR1* (rs200563280 C > T and rs199895006 C > T); ‘drug response’ in *CYP2D6* (rs1058172 C > T) and *ABCB1* (rs201122883 G > A); and ‘Pathogenic’ in *CYP1B1* (rs377049098 G > A), *XPC* (rs121965088 G > A), and *CFTR* (rs397508198 G > T and rs74597325 C > T).

## Discussion

In this study, we conducted an in-depth analysis of population-level PGx variation and phenotype landscape using WGS data from the 1KGP and a reliable SV-aware method, PyPGx. Overall, (1) we identified global haplotype and phenotype frequencies by including SVs for seven and ten pharmacogenes, respectively, (2) we validated PyPGx via detection of known SVs and identified novel SVs, including SVs that have not been used to define star alleles in this well-characterized dataset, (3) most of the cohort (98.2%) had at least one non-typical drug response according to CPIC guidelines, consistent with previous findings^11^, and (4) we identified a large number of variants (210 SNVs and indels) not previously used to define star alleles that were potentially deleterious with high CADD scores and clinical significance. These findings contribute to an enhanced understanding of global PGx variation.

The precise determination of haplotype and phenotype frequencies across populations is crucial for enhancing drug selection and dosage accuracy, with the potential to mitigate adverse drug events, though we acknowledge that these frequencies are generalizations of diverse communities, and that individualized PGx testing is optimal for personalizing care. While we have successfully updated frequencies for specific examples, there remains a gap in reporting SVs comprehensively across all populations, presenting challenges for meaningful comparisons. One of the most comprehensive studies of PGx variation to date^11^ excluded SVs analyses. Further, our study used high-coverage WGS data, in contrast to the previous study’s use of imputed, exome, and integrated datasets. Another recent study^18^, using WGS data, conducted a thorough examination of coding and non-coding SVs influencing drug absorption, distribution, metabolism, and excretion. While the study was comprehensive^18^, PyPGx adds another dimension by facilitating the detection of “novel” SVs, those not currently used to define star alleles. Our study demonstrates the utility of PyPGx and will significantly enhance the scientific communities’ ability to accurately detect and measure SV frequencies.

PyPGx’s capacity to detect SVs also enables the correction of misidentified haplotype calls, particularly for complex, polymorphic pharmacogenes, such as *CYP2D6*. While *CYP2D6* genotypes are well studied, many haplotype frequencies may exclude SVs. Using our pipeline, we were able to enhance *CYP2D6* haplotype calls containing SVs in 1KGP, including: *1 × *2*, *4 × *2*, *2 × *2*, *5, *10 × *2* (Supplementary Table 5). While beyond the scope of this paper, a more comprehensive exploration of the biological and PGx impacts of SVs is needed.

We also examined variants that had not been used to define star alleles to determine if any were clinically relevant. Notably, we identified 8 variants with previous reports, including a nonsense variant within *CFTR* (rs74597325), which had the most journal submissions (n = 26) with the highest review status (4/4; practice guidelines), as well as pathogenic implications in cystic fibrosis^51^. In addition, a nonsense variant within *RYR1* (rs200563280) had the next highest amount of evidence (n = 20) of pathogenicity and likely pathogenicity, with association with malignant hyperthermia susceptibility^52^. Both variants have yet to be utilized in defining a star allele. However, the rest of the variants had less supporting evidence and publications (n < 6).

While our work has numerous strengths, including population-level comparisons, SV analyses, and an exploration of potentially clinically significant variants, it is not without limitations. Both the 1KGP and PharmGKB grouping systems rely on genetic similarity; however, due to differences in the grouping system and potential sampling bias, the comparisons may be indirect or incomplete. 1KGP-SAS, 1KGP-EAS, and 1KGP-EUR are consistent with PharmGKB; however, 1KGP-AMR and 1KGP-AFR are not fully consistent ^31^; findings are suggested to be viewed as approximations^31^. These population descriptors are used to enhance comparability and reproducibility, but are limited. Notably, 1KGP-SSA is underrepresented within the dataset^53^. While PyPGx cannot detect breakpoints of SVs, applying machine-learning algorithms, like SVM, has helped to improve identification of SVs and enhances our understanding of the complexity of variation within pharmacogenes. We also encountered “extreme” fold changes when comparing haplotype frequencies (N = 10), while most could be explained by low frequencies (N = 5/10) and study outliers within PharmGKB (N = 3/10), we encountered “extreme” instances with *DPYD* specifically (N = 2/10) due to technical aspects of PyPGx and the PharmVar star allele definition of *DPYD c.1896T* > *C* haplotype, which results in normal function, but is not considered a reference haplotype. Additionally, PyPGx detected frequencies for haplotypes, including those containing SVs, and phenotypes (e.g., “Likely” or “Possible” status) were not available for comparison within PharmGKB. Yet, this does highlight that PyPGx enables us to expand upon our current characterization of haplotype and phenotype frequencies. Ultimately, while most of our haplotype and phenotype calls are largely consistent with existing literature, we were able to offer valuable population-level insights into relevant haplotype and phenotypes, while incorporating SVs.

The utilization of high-resolution WGS data significantly enhances our comprehension of PGx variation across populations and has the potential to optimize precision medicine; yet, further efforts are needed to ensure all receive equitable benefits of PGx research^54^. Further, individual testing is optimal for precision medicine efforts. Potential future directions of this work include functional validation studies to validate findings, especially of the 8 variants identified, as well as assays to confirm phenotypic predictions across populations; however, that is outside the scope of this work. Additionally, the methods used here could be implemented in larger cohorts, such as the UK Biobank, which recently released WGS data for all 500,000 participants. In summary, our study expands upon similar efforts^14^ and stands as a valuable resource on global PGx variation and underscores the potential of WGS data to play a pivotal role in advancing precision medicine.

## Supplementary Information

Supplementary Information 1.

Supplementary Information 2.

Supplementary Information 3.

Supplementary Information 4.

Supplementary Information 5.

Supplementary Information 6.

Supplementary Information 7.

Supplementary Information 8.

Supplementary Information 9.

Supplementary Information 10.

Supplementary Information 11.

Supplementary Information 12.

## Data Availability

Code and corresponding data supporting the current study is available at: https://github.com/sbslee/1kgp-pgx-paper.
