# Supplementary figures and images for: Pharmacogenetic analysis of structural variation in the 1000 genomes project using whole genome sequences
Source: Sci Rep. 2024 Oct 1;14:22774. doi: 10.1038/s41598-024-73748-3 (PMC11445439; doi:10.1038/s41598-024-73748-3)

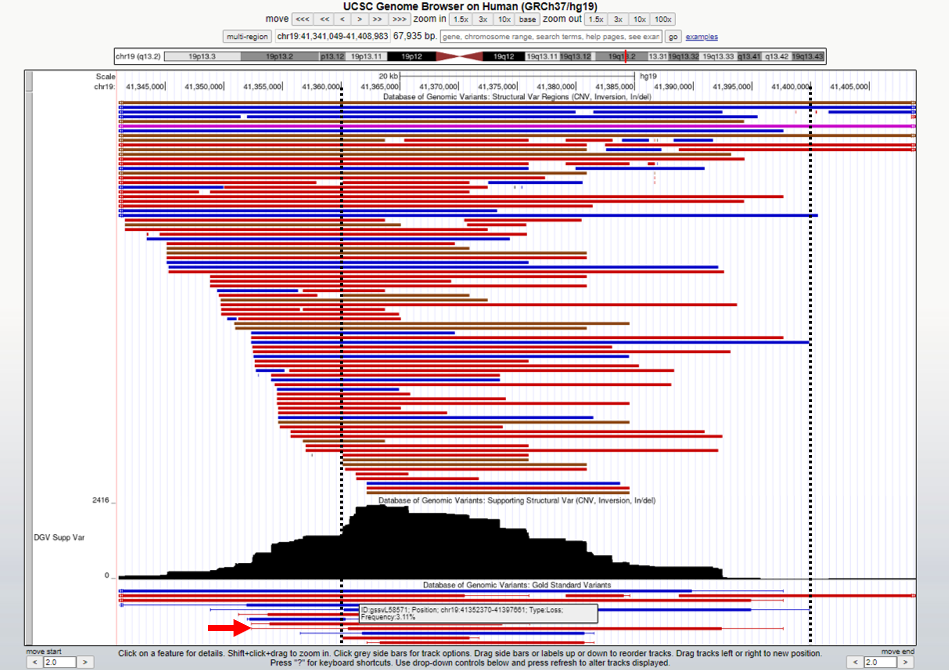

Supplement: Supplementary file 1 — Supplementary Information 1. [file 41598_2024_73748_MOESM1_ESM.png]

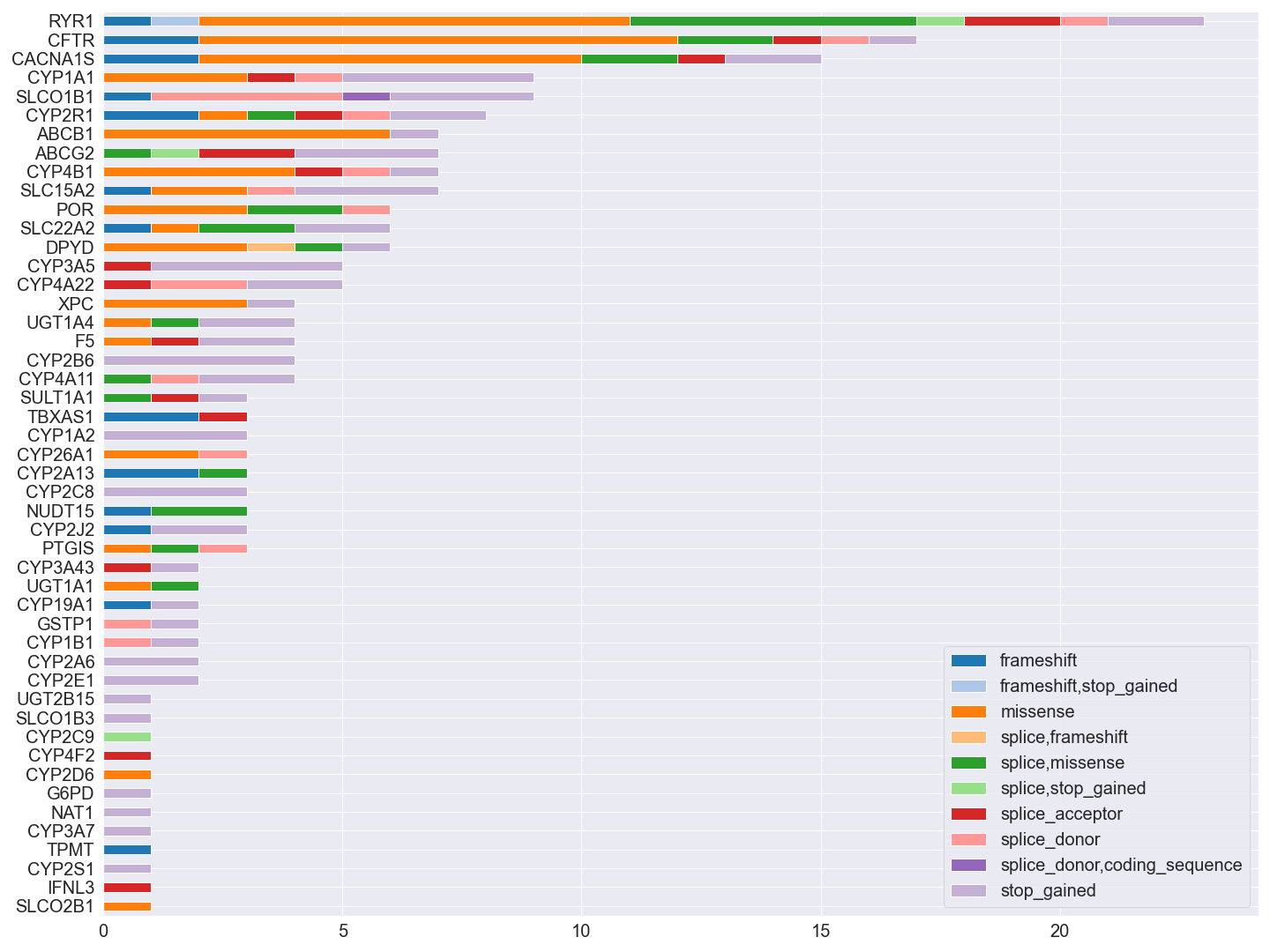

Supplement: Supplementary file 2 — Supplementary Information 2. [file 41598_2024_73748_MOESM2_ESM.png]
