# Supplementary material for: Pharmacogenetic analysis of structural variation in the 1000 genomes project using whole genome sequences
Source: Sci Rep. 2024 Oct 1;14:22774. doi: 10.1038/s41598-024-73748-3 (PMC11445439; doi:10.1038/s41598-024-73748-3)
Supplement: Supplementary file 4 — Supplementary Information 4. [file 41598_2024_73748_MOESM4_ESM.pdf]

Gene	Train						Test					
	Before		After		Accuracy		Before		After		Accuracy	
	Size	Unique	Size	Unique	GRCh37	GRCh38	Size	Unique	Size	Unique	GRCh37	GRCh38
CYP2A6	108	6	429	22	1	1	27	4	88	22	1	1
CYP2B6	50	2	100	7	1	1	21	2	26	7	1	1
CYP2D6	245	11	734	19	0.99	0.992	61	11	167	19	1	1
CYP2E1	70	4	278	9	1	1	24	4	67	9	1	1
CYP4F2	55	2	58	2	1	1	21	2	21	2	1	1
GSTM1	56	4	253	9	1	1	20	4	73	9	1	1
SLC22A2	54	3	143	6	1	1	22	3	34	6	1	1
SULT1A1	231	5	610	10	0.997	0.997	63	5	149	10	1	1
UGT1A4	52	3	97	5	1	1	20	1	27	5	1	1
UGT2B15	55	2	126	9	1	1	20	1	30	9	1	1
UGT2B17	50	3	249	8	1	1	20	3	56	8	1	1
